# Impact of Premature Ventricular Complex Burden on Ischemic Stroke in Patients with Non-Valvular Atrial Fibrillation

**DOI:** 10.3390/jcm13175009

**Published:** 2024-08-24

**Authors:** Juwon Kim, Ju Youn Kim, Seung-Jung Park, Young Keun On, Kyoung-Min Park

**Affiliations:** Division of Cardiology, Department of Medicine, Heart Vascular Stroke Institute, Samsung Medical Center, Sungkyunkwan University School of Medicine, Seoul 06351, Republic of Korea; abcd186a@gmail.com (J.K.); kzzoo921@gmail.com (J.Y.K.); orthovics@gmail.com (S.-J.P.); yk.on@samsung.com (Y.K.O.)

**Keywords:** premature ventricular complex, atrial fibrillation, stroke, heart failure

## Abstract

**Background/Objectives**: Limited data are available regarding the prognostic impact of premature ventricular complex (PVC) burden in patients with atrial fibrillation (AF). We sought to compare clinical outcomes in patients with AF according to PVC burden via 24 h Holter monitoring. **Methods**: From January 2010 to December 2020, 4834 oral anticoagulant (OAC)-naïve non-valvular AF (NVAF) patients who underwent 24 h Holter monitoring were included for analysis. **Results**: Among the 4834 OAC-naïve NVAF patients, 2835 patients (58.6%) exhibited at least one PVC within a 24 h monitoring period, and 120 patients (2.5%) displayed a daily PVC burden exceeding 10%. In the follow-up echocardiography, patients with a daily PVC burden of ≥10% exhibited lower left ventricular ejection fraction, larger left atrial volume, and higher right ventricular systolic pressure and E/e’ than those with a daily PVC burden of <10%. The risk of ischemic stroke (adjusted HR 2.332, *p* = 0.015) and heart failure admission (adjusted HR 2.147, *p* = 0.010) were significantly higher in the patients with a daily PVC burden of ≥10% than in those with a daily PVC burden of <10%. However, the incidence of cardiac death was not significantly different between the two groups. A daily PVC burden of ≥10% was independently associated with the risk of ischemic stroke in the OAC-naïve NVAF patients, irrespective of the CHA_2_DS_2_–VASc score, AF type, and left atrial size. **Conclusions**: The current results suggest that evaluating and monitoring the burden of PVCs in patients with NVAF is an important aspect of predicting stroke and heart failure admission.

## 1. Introduction

The focus of the management of patients with atrial fibrillation (AF) is stroke prevention due to its connection with a fivefold increase in the risk of stroke [1]. However, this risk is not consistent and varies based on the presence of other specific factors or modifiers that influence the risk of stroke. The current guidelines recommend the assessment of stroke risk using the CHA_2_DS_2_–VASc score in patients with AF [1,2]. However, this score does not incorporate all risk factors contributing to thromboembolic events [3,4].

Premature ventricular complex (PVC) is one of the most common arrhythmias frequently encountered in clinical situations. The connection between high PVC burden and heightened risk of cardiomyopathy has been established [5,6]. The mitigation of PVCs results in the enhancement of left ventricular function. Interestingly, some studies have demonstrated that PVCs are associated with an increased risk of AF and ischemic stroke [7,8,9,10,11,12]. However, most of these studies examined the correlation between PVCs and clinical outcomes, including ischemic stroke, in patients who had not been previously diagnosed with AF.

To our knowledge, the relationship between high PVC burden and increased risk of ischemic stroke in patients with AF has not been investigated. Therefore, our study sought to assess the correlation between elevated PVC burden and alterations in echocardiographic measurements and clinical outcomes in patients with AF using 24 h Holter electrocardiography (ECG).

## 2. Materials and Methods

### 2.1. Study Population

**Figure 1:**
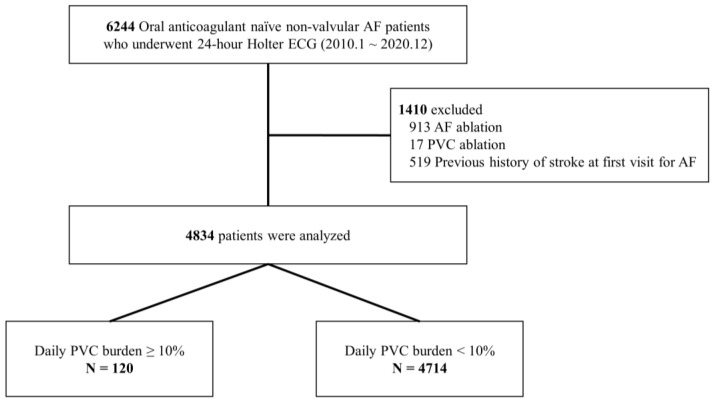
Study Flow. AF, atrial fibrillation; ECG, electrocardiography; PVC, premature ventricular complex.

We screened 6244 oral anticoagulant (OAC)-naïve non-valvular AF (NVAF) patients who expressed documented AF on a 12-lead ECG and underwent 24 h Holter ECG at our institute between January 2010 and December 2020. Patients who underwent AF catheter ablation (n = 913) or PVC ablation (n = 17) during the study period and those with a previous history of stroke at first visit for AF (n = 519) were excluded. The remaining 4834 OAC-naïve NVAF patients who had no history of stroke were selected for the current study (Figure 1). The Institutional Review Board of Samsung Medical Center approved this study and waived the requirement for written informed consent. The study protocol complied with the guidelines of the Declaration of Helsinki.

### 2.2. Data Collection and Study Outcomes

Baseline characteristics data, including age, sex, anti-arrhythmic drug usage, and the presence of cardiovascular risk factors, were obtained from the electronic medical record database of our hospital using a data-mining system, enabling the retrieval of relevant information for the study. The cardiovascular risk factors assessed included the presence of comorbidities, diabetes, hypertension, chronic kidney disease, and peripheral vascular disease; a history of myocardial infarction; and a history of heart failure, with a left ventricular ejection fraction (LVEF) of less than 50%. AF was considered persistent if the AF was sustained for at least one week.

PVC burden was evaluated utilizing a three-channel 24 h Holter device. All 24 h Holter data during the follow-up period were collected for analysis, and changes in PVC burden were evaluated. The results of 24 h Holter testing were validated by two separate cardiologists, ensuring its accuracy and reliability. To differentiate between a PVC and an aberrantly conducted beat, pre-defined criteria were used (Supplementary Table S1). In cases in which multiple 24 h Holter studies were conducted on the same patient, the index 24 h Holter study was the one that exhibited the highest PVC burden. To classify the type of PVCs, structural heart diseases were defined as myocardial infarction, heart failure, sustained ventricular tachycardia, survived cardiac arrest, valvular heart disease (severe aortic stenosis, severe mitral regurgitation, and mitral valve prolapse), previous cardiac surgery, or percutaneous revascularization [13].

Baseline echocardiographic findings were assessed based on the most recently performed echocardiography within a year prior to the first AF visit. If not available, as most second visits occur within 2 months, baseline data were obtained from echocardiography performed within 2 months following the first visit for AF. Because echocardiographic examinations during follow-up were not standardized across physicians, follow-up echocardiographic parameter data were obtained from the most recent echocardiography conducted during the follow-up period. Left atrial (LA) volume was measured using the biplane area-length method, and the LA volume index (LAVI) was derived by dividing the LA volume by the body surface area. LVEF was assessed using Simpson’s biplane method.

The endpoints were ischemic stroke, death from cardiac-related causes, and heart failure (HF) admission after the initial visit for AF management. Thrombotic stroke included lacunar stroke, stroke attributable to small vessel disease, and stroke caused by large artery atherosclerosis. Embolic stroke included cardioembolic stroke and embolic stroke of undetermined source (non-lacunar on neuroimaging, without an obvious embolic source after evaluation) [14]. Two separate cardiologists validated the endpoints, and a third cardiologist adjudicated in cases of disagreement. In order to eliminate the effect of OAC treatment on the study outcomes, patients were censored if they were prescribed OACs after study entry.

### 2.3. Statistical Analysis

Continuous variables were analyzed using the unpaired *t*-test or the Mann–Whitney rank-sum test and are presented as mean and standard deviation or median with interquartile range, according to their distributions. These distributions underwent Kolmogorov–Smirnov testing, and the Q–Q plots were visually inspected. All discrete and categorical variables are presented as numbers and relative frequencies (percentages) and were compared using the Chi-square test or Fisher’s exact test.

The cumulative incidences of ischemic stroke, cardiac-related death, and HF admission are presented as Kaplan–Meier estimates and are compared using a log-rank test or a Breslow test. A multivariable Cox proportional hazard regression was used to calculate hazard ratios (HRs) and 95% confidence intervals (CIs) to compare the risk of clinical events according to the PVC burden. The assumption of proportionality was assessed by the Schoenfeld residuals and graphically, by the log–log plot. Multivariable Cox proportional hazard models were constructed using clinically relevant variables to identify independent predictors of ischemic stroke. All analyses were two-tailed, and statistical significance was defined as *p* < 0.05. Statistical analyses were performed using SPSS 25.0 for Windows (SPSS-PC, Chicago, IL, USA) and R version 3.6.0 (R Foundation for Statistical Computing, Vienna, Austria).

## 3. Results

### 3.1. PVC Burden and Baseline Characteristics

Among the 4834 OAC-naïve NVAF patients, 2835 (58.6%) experienced at least one PVC during the 24 h Holter testing period, and 120 (2.5%) displayed a daily PVC burden of over 10% (Supplementary Table S2). The patients were divided into two groups: a high PVC burden group [daily PVC burden ≥ 10%, n = 120 (2.5%)] and a low PVC burden group [daily PVC burden < 10%, n = 2399 (97.7%)]. The PVC burden cut-off point was set at 10%, based on previous studies demonstrating that PVC burden is associated with an increased risk of cardiomyopathy [5,6,15,16]. The baseline patient characteristics in the high and low PVC burden groups are presented in Table 1. Patients in the high PVC burden group were more likely to be women; had a higher prevalence of diabetes, hypertension, previous myocardial infarction, and heart failure; and had higher CHA_2_DS_2_–VASc scores compared to those in the low PVC burden group. Class III anti-arrhythmic agents and beta blockers were more frequently used in the high PVC burden group.

The type and locations of PVCs are presented in Table 1. The majority of cases had an idiopathic cause, and the location of PVCs was comparable between the two groups. PVCs from an outflow tract were more frequent than those from a non-outflow tract. Structural heart disease was present in 17.1% of cases. Non-ischemic PVCs were more frequently observed than were ischemic PVCs.

### 3.2. Echocardiographic Parameters According to PVC Burden

Table 2 shows the baseline and follow-up echocardiographic parameters according to PVC burden. Compared with patients with a daily PVC burden of <10%, those with a daily PVC burden of ≥10% displayed a lower LVEF on the baseline echocardiography. At the follow-up echocardiography, patients with a daily PVC burden of ≥10% showed a lower LVEF, a larger LA size, and a higher RVSP and E/e’ than those with a daily PVC burden of <10%. The time from baseline echocardiography to follow-up echocardiography was similar between the two groups (median of almost 3 years). The ΔLVEF, ΔLA volume index, ΔLA diameter, and ΔE/e’ from baseline were significantly different between the two groups.

### 3.3. Clinical Outcomes according to PVC Burden

**Figure 2:**
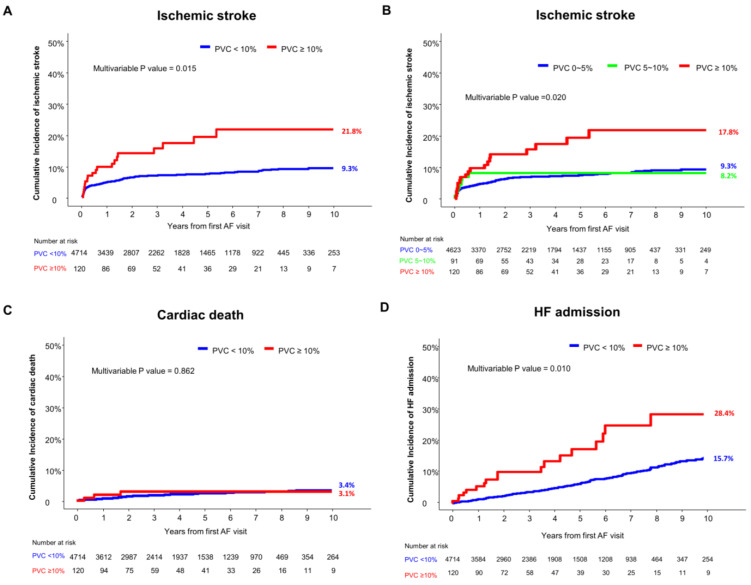
Cumulative incidence of clinical outcomes according to daily PVC burden in OAC-naïve NVAF patients. (**A**) Cumulative incidence of ischemic stroke in patients with daily PVC burden of ≥10% and those with daily PVC burden of <10%; (**B**) cumulative incidence of ischemic stroke in patients with daily PVC burden of ≥10%, those with daily PVC burden of 5 to 10%, and those with daily PVC burden < 5%; (**C**) cumulative incidence of cardiac death; (**D**) cumulative incidence of HF admission in patients with daily PVC burden of ≥10% and those with daily PVC burden of <10%. AF, atrial fibrillation; HF, heart failure; OAC, oral anticoagulant; NVAF, non-valvular atrial fibrillation; PVC, premature ventricular complex.

The median follow-up duration of the study population was 1611 days (interquartile range: 746–2649 days). In the 4834 OAC-naïve NVAF patients, subjects with a daily PVC burden of ≥10% showed a significantly higher risk of ischemic stroke than those with a daily PVC burden of <10% (21.8% vs. 9.3%, adjusted HR 2.332, 95% CI 1.179–4.614, *p* = 0.015) (Table 3 and Figure 2A). The incidences of embolic stroke were significantly different between the two groups. But, the incidences of thrombotic stroke were similar between the two groups. The incidence of ischemic stroke was significantly higher in the patients with a daily PVC burden of ≥10%, but was comparable between the patients with a daily PVC burden of <5% and those with a daily PVC burden of 5 to 10% (Figure 2B).

In the 4834 OAC-naïve NVAF patients, a daily PVC burden of ≥10% was independently associated with ischemic stroke after adjusting for CHA_2_DS_2_–VASc score, AF type, antiarrhythmic agent usage, PVC location, LVEF, LA volume index, and E/e’ (Table 4). The risk of ischemic stroke was not significantly different in regards to the PVC location (Supplementary Figure S1).

The incidence of cardiac-related death was not significantly different between the patients with a daily PVC burden of ≥ 10% and those with a daily PVC burden of <10% (Table 3 and Figure 2C). The risk of HF admission was significantly higher in the high PVC burden group than in the low PVC burden group (Table 3 and Figure 2D).

**Figure 3:**
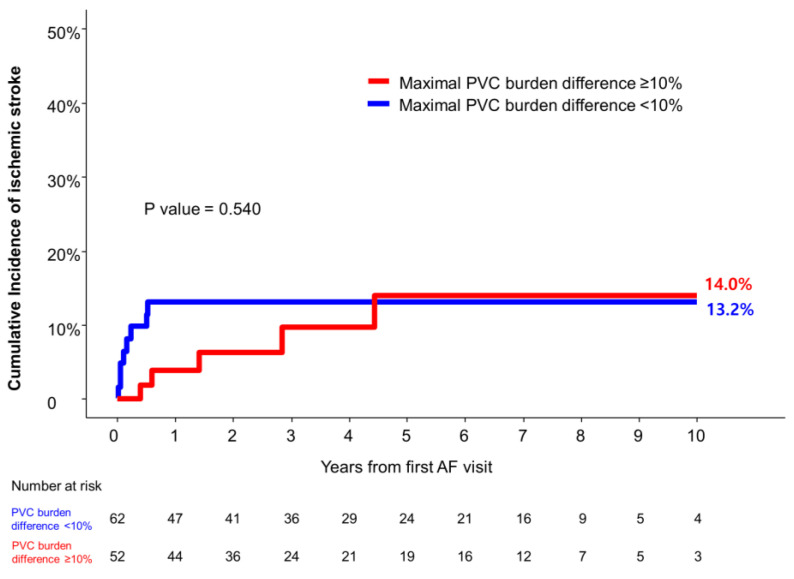
Cumulative incidence of ischemic stroke according to the maximal difference of daily PVC burden between 24 h Holter studies. Cumulative incidence of ischemic stroke between the patients with maximal PVC burden difference of ≥10% and those with maximal PVC burden difference of <10% between 24 h Holter studies. AF, atrial fibrillation; PVC, premature ventricular complex.

Among the patients who showed a daily PVC burden of ≥10% with multiple 24 h Holter studies (n = 114), the cumulative incidences of ischemic stroke did not differ significantly between the patients with a maximal PVC burden difference of ≥10% and those with a maximal PVC burden difference of <10% between the 24 h Holter studies (*p* = 0.540) (Figure 3).

## 4. Discussion

The present study evaluated the clinical implications of PVC burden in OAC-naïve NVAF patients. Two major findings emerged. First, the patients with a daily PVC burden of ≥10% had a significantly higher risk of ischemic stroke and HF admission than the patients with a daily PVC burden of <10%. Second, a daily PVC burden of ≥10% was independently associated with the risk of ischemic stroke in the OAC-naïve NVAF patients, irrespective of CHA_2_DS_2_–VASc score, AF type, antiarrhythmic agent usage, PVC location, LVEF, LA volume index, and E/e’.

### 4.1. PVC Burden and Stroke in Patients with AF

Some previous studies reported that the presence of PVCs may increase the risk of ischemic stroke [7,9,12]. However, those studies had not investigated the relationship between PVC burden and the risk of ischemic stroke, and patients with AF either comprised a small proportion of the overall study population or were excluded. To the best our knowledge, the present study was the first to suggest that a higher PVC burden in AF patients is associated with an increased risk of ischemic stroke.

There are several plausible explanations for the current results. First, the presence of a high PVC burden may result in a decline in LV systolic function, potentially contributing to the occurrence of ischemic stroke. The association between an elevated PVC burden and an increased susceptibility to cardiomyopathy has been well-established [5,6,15,16]. Specifically, there were no instances of cardiomyopathy observed when the PVC burden was below 10%. Similarly, in the current study, a PVC burden below 10% among patients with AF was not clinically significant. Despite falling within the normal range, patients with a daily PVC burden ≥ 10% exhibited significantly lower LVEF on follow-up echocardiography compared to those with a daily PVC burden of <10%. Notably, PVC burden displays substantial fluctuations, and the clinical significance of these changes in burden has not been clearly elucidated [16,17]. In the current study, variations in the PVC burden over time did not appear to impact the incidence of ischemic stroke. This implies that the risk of ischemic stroke can be elevated among patients with AF who have a PVC burden exceeding 10%, even on a single occasion. Nevertheless, the limited sample size of patient data analyzed may have contributed to the lack of significant findings. Second, the elevation of LV filling pressure caused by a significant PVC burden might result in atrial stretch and atrial cardiomyopathy, ultimately contributing to the development of ischemic stroke. Indeed, in the follow-up echocardiography, patients with a daily PVC burden of ≥10% showed indications of poorer diastolic function in regards to LA size, RVSP, and E/e’ than did those with a daily PVC burden of <10%. Some studies demonstrated that an enlarged LA and increased diastolic dysfunction are associated with arrhythmic burden and ischemic stroke in AF patients [3,18,19,20]. Third, the presence of a significant PVC burden could potentially contribute to an increased AF burden. Previous studies have documented a correlation between PVC and new-onset AF, indicating a potential mechanism involving retrograde atrial activation and the development of atrial cardiomyopathy. Some studies reported electrophysiologic characteristics predicting the development of AF [21]. Additionally, there was an independent association between a higher total AF burden and increased rates of thromboembolic events [22,23,24].

Considering these potential explanations, the current results suggest that the presence of PVCs in patients with NVAF could be a factor in assessing the risk of stroke, and that evaluating and monitoring the burden of PVCs is an important aspect of predicting stroke. The patients with a high PVC burden can be successfully treated with catheter ablation, indicating that PVCs could potentially be a modifiable risk factor for ischemic stroke in patients with NVAF. In a previous study, catheter ablation for high PVC burden showed a trend in decreasing the development of new-onset AF [10]. In the present study, among 6244 patients, 95 had a daily PVC burden exceeding 15%, of whom 17 (17.9%) underwent PVC ablation. The relatively low rate of PVC ablation is not only associated with the strict and limited financial reimbursement criteria in Korea, but also with the conservative Asian attitudes toward procedures. Further studies are needed to investigate the incorporation of high PVC burden into thromboembolic risk models and to assess the effectiveness of catheter ablation for PVCs in preventing strokes among patients with NVAF.

### 4.2. Study Limitations

Some limitations of the study should be acknowledged. First, the study was a non-randomized, observational study. To overcome potential biases, we performed model-based adjustment, but unmeasured factors might have affected the outcomes. Second, there was a substantial disparity in population size between the two groups, and the number of individuals in the high PVC burden group was limited. Third, antiarrhythmic agent usage and change might have affected the results of the current study. Fourth, we did not have direct access information regarding AF duration. Fifth, PVC burden might be underestimated in patients who undergo a limited number of 24 h Holter tests during follow-up. The ECG patch for long-term monitoring was first introduced in Korea in 2019 and has been widely used since mid-2020, after the resolution of insurance-related issues. Unfortunately, this study was conducted on patients from 2010 to 2020, limiting the analysis of VPC burden using long-term ECG patch monitoring. Sixth, 736 patients with a CHA_2_DS_2_–VASc score greater than 2 did not receive OACs during the study period (33 patients in the PVC burden ≥ 10% group and 703 patients in the PVC burden < 10% group). The primary reason for not prescribing OACs was the presence of high bleeding risk (such as a history of major bleeding or conditions with an elevated bleeding risk). Withholding anticoagulation therapy without valid reasons in patients with a CHA_2_DS_2_–VASc score above 2 could raise ethical concerns. Therefore, to test our hypothesis, a prospective study, particularly a randomized controlled trial, would not be feasible. Consequently, the study could only be conducted using a retrospective observational design. In this context, the potential ethical implications paradoxically underscore the unique advantage of our study design.

## 5. Conclusions

In OAC-naïve NVAF patients without prior stroke, high daily PVC burden (≥ 10%) was independently associated with a higher risk of ischemic stroke, irrespective of CHA_2_DS_2_–VASc score, AF type, antiarrhythmic agent usage, PVC location, LVEF, LA volume index, and E/e’ during long-term follow-up.

## Figures and Tables

**Table 1:** Baseline characteristics of the study population according to daily PVC burden.

	PVC Burden ≥ 10%	PVC Burden < 10%	*p* Value
	N = 120 (2.5)	N = 4714 (97.5)	
Demographics			
Age, years	64.1 ± 13.1	62.2 ± 12.9	0.113
Male sex	71 (59.2)	3221 (68.3)	0.043
Cardiovascular risk factors			
Diabetes mellitus	17 (14.2)	392 (8.3)	0.035
Hypertension	54 (45.0%)	1054 (22.4)	<0.001
Chronic kidney disease	5 (4.2)	128 (2.7)	0.498
Peripheral vascular disease	1 (0.8)	55 (1.2)	1.000
Previous myocardial infarction	12 (10.0)	147 (3.1)	<0.001
Heart failure	22 (18.3)	235 (5.0)	<0.001
CHA_2_DS_2_–VASc score	2.2 ± 1.4	1.3 ± 1.3	<0.001
Type of AF			0.993
Paroxysmal	104 (86.7)	4064 (86.2)	
Persistent	16 (13.3)	650 (13.8)	
Antiarrhythmic agent usage			
Class Ic	30 (25.0)	1169 (24.8)	1.000
Class III	43 (35.8)	1220 (25.9)	0.019
Beta blocker	65 (54.2)	1918 (40.7)	0.004
Calcium channel blocker	12 (10.0)	704 (14.9)	0.170
PVC burden during 24 h	21.8 ± 13.8	0.3 ± 1.1	<0.001
PVC type	N = 120	N = 560	0.781
Idiopathic	95 (79.2)	469 (83.8)	
RVOT	35 (29.2)	177 (31.6)	
LVOT	34 (28.3)	157 (28.0)	
Non-OT	26 (21.7)	135 (24.1)	
Structural heart disease	25 (20.8)	91 (16.3)	
Non-ischemic	16 (13.3)	61 (10.9)	
Ischemic	9 (7.5)	30 (5.4)	

Values are presented as the mean ± standard deviation, median (interquartile range), or number (%). AF, atrial fibrillation; LVOT, left ventricular outflow tract; OT, outflow tract; PVC, premature ventricular complex; RVOT, right ventricular outflow tract.

**Table 2:** Baseline and follow-up echocardiogram according to daily PVC burden.

	PVC Burden ≥ 10%	PVC Burden < 10%	*p* Value
	N = 120 (2.5)	N = 4714 (97.5)	
Baseline echocardiogram			
LV ejection fraction (%)	57.9 ± 10.9	61.2 ± 9.2	0.004
LA volume index (mL/m^2^)	47.4 ± 20.2	44.8 ± 19.6	0.196
LA diameter (mm)	44.1 ± 7.7	42.7 ± 7.9	0.064
RV systolic pressure (mmHg)	30.0 ± 8.7	28.4 ± 7.9	0.091
E/e’	11.1 ± 4.7	10.1 ± 5.1	0.084
Follow-Up echocardiogram			
Time to follow-up echocardiogram (day)	1179 (533–2226)	1095 (261–2078)	0.242
LV ejection fraction (%)	55.3 ± 13.8	61.2 ± 8.7	<0.001
LA volume index (mL/m^2^)	51.9 ± 24.5	46.8 ± 21.5	0.025
LA diameter (mm)	45.5 ± 7.7	43.0 ± 8.0	0.003
RV systolic pressure (mmHg)	31.2 ± 8.3	29.1 ± 8.7	0.042
E/e’	13.0 ± 7.8	10.5 ± 5.5	0.004
ΔLV ejection fraction from baseline (%)	−1.8 (−9.0–4.0)	0.0 (−6.0–6.0)	0.038
ΔLA volume index from baseline (mL/m^2^)	5.8 (−1.5–13.8)	2.0 (−6.3–11.6)	0.039
ΔLA diameter from baseline (mm)	2.8 (−1.6–5.1)	0.4 (−3.1–4.0)	0.036
ΔRV systolic pressure from baseline (mmHg)	0.2 (0.0–3.8)	0.0 (−0.2–2.8)	0.227
ΔE/e’ from baseline	1.0 (−0.5–4.2)	0.3 (−1.7–2.4)	0.036

Values are presented as the mean ± standard deviation and median (interquartile range). LA, left atrium; LV, left ventricle; PVC, premature ventricular complex; RV, right ventricle.

**Table 3:** Clinical outcomes after first AF visit according to PVC burden.

	PVC Burden ≥ 10%	PVC Burden < 10%	Univariable HR (95% CI)	Multivariable HR (95% CI) *	*p* Value
	N = 120	N = 4714			
Ischemic stroke	19 (21.8%)	304 (9.3%)	2.469 (1.553–3.952)	2.332 (1.179–4.614)	0.015
Embolic stroke	17 (20.1%)	229 (7.0%)	2.937 (1.794–4.807)	3.370 (1.606–7.071)	0.001
Thrombotic stroke	2 (2.2%)	75 (2.5%)	1.048 (0.257–4.269)	1.111 (0.271–4.561)	0.883
Cardiac death	3 (3.1%)	82 (3.4%)	1.399 (0.442–4.429)	0.903 (0.284–2.873)	0.862
HF admission	18 (28.4%)	253 (15.7%)	2.733 (1.694–4.409)	2.147 (1.197–3.848)	0.010

The cumulative incidences of clinical outcomes are presented as event number and Kaplan–Meier estimates from initial AF visit. *p* values were derived from a multivariable Cox regression model. * Covariables which were included in the multivariable adjusted Cox regression model were diabetes, hypertension, previous myocardial infarction, heart failure, CHA_2_DS_2_–VASc score, type of AF, antiarrhythmic agent usage, type of PVC, LV ejection fraction, and LA volume index. AF, atrial fibrillation; CI, confidence intervals; HF, heart failure; HR, hazard ratios; LA, left atrium; LV, left ventricle; PVC, premature ventricular complex.

**Table 4:** Independent predictors for ischemic stroke in OAC-naïve NVAF patients.

Variable	Univariate Analysis		Multivariate Analysis *	
	HR (95% CI)	*p* Value	HR (95% CI)	*p* Value
PVC burden ≥ 10%	2.469 (1.553–3.952)	<0.001	3.508 (1.671–7.363)	<0.001
CHA_2_DS_2_–VASc score, per 1 score increase	1.321 (1.227–1.423)	<0.001	1.488 (1.132–1.957)	0.004
Persistent AF	2.253 (1.738–2.921)	<0.001	3.467 (0.932–12.899)	0.064
Antiarrhythmic agent usage	0.910 (0.720–1.150)	0.427	0.607 (0.284–1.299)	0.199
PVC location (non-OT)	0.933 (0.646–1.348)	0.713	1.193 (0.761–1.872)	0.441
LV ejection fraction, per 1% increase	0.994 (0.983–1.006)	0.335	1.021 (0.980–1.065)	0.320
LA volume index, per 1 mL/m^2^ increase	1.011 (1.006–1.016)	<0.001	1.009 (0.987–1.033)	0.413
E/e’, per 1 increase	1.030 (1.014–1.046)	<0.001	1.010 (0.919–1.110)	0.830

* Discriminant ability of multivariable model was 0.673 (95% CI 0.581–0.674). AF, atrial fibrillation; HR, hazard ratios; LA, left atrium; LV, left ventricle; OT, outflow tract; PVC, premature ventricular complex.

## Data Availability

The data that support the finding of this study are available from the corresponding author upon reasonable request.

## Supplementary Materials

The following supporting information can be downloaded at: https://www.mdpi.com/article/10.3390/jcm13175009/s1, Figure S1: Cumulative incidence of ischemic stroke according to PVC location in OAC-naïve NVAF patients; Table S1: The criteria to differentiate between a PVC and an aberrantly conducted beat; Table S2: PVC burden in OAC-naïve NVAF patients.
