# Supplementary material for: Impact of Premature Ventricular Complex Burden on Ischemic Stroke in Patients with Non-Valvular Atrial Fibrillation
Source: J Clin Med. 2024 Aug 24;13(17):5009. doi: 10.3390/jcm13175009 (PMC11396718; doi:10.3390/jcm13175009)
Supplement: Supplementary file 1 [file jcm-13-05009-s001.zip › jcm-3142451-supplementary.pdf]

Supplementary Materials

Contents

Supplementary Table S1. The criteria to differentiate a PVC and an aberrantly conducted beat.

Supplementary Table S2. PVC burden in OAC-naïve NVAF patients.

Supplementary Figure S1. Cumulative incidence of ischemic stroke according to PVC Location in OAC-naïve NVAF patients.

Supplemental Table S1. The criteria to differentiate a PVC and an aberrantly conducted beat

If the criteria were met, the beat was classified as a PVC; otherwise, it was classified as an aberrantly conducted beat.

Criteria
1) Absence of preceding ectopic P wave in the Holter or 12 lead ECG
2) Atypical QRS morphologies in the Holter or 12 lead ECG
- If RBBB morphology, monophasic R waves or R waves with a notch on the down-stroke of the R waves in V1, or R/S ratio < 1 in V6
- If LBBB morphology, R wave > 0.04 sec, a notch on down-stroke of S wave, or > 0.06 sec delay from QRS onset to nadir of S wave in V1, or monophasic QS in V6
3) Complete compensatory pause in the Holter or 12 lead ECG

Supplemental Table S2. PVC burden in OAC-naïve NVAF patients

PVC burden during 24 hours	Patients number
**Total**	**N=4834**
No PVC	1999 (41.4%)
0 < PVCs < 1%	2395 (49.5%)
1 ≤ PVCs <5%	229 (4.7%)
5 ≤ PVCs <10%	91 (1.9%)
10 ≤ PVCs < 20%	71 (1.5%)
20% ≤ PVCs	49 (1.0%)

Values are presented as number (%).

NVAF, non-valvular atrial fibrillation; OAC, oral anticoagulant; PVC, premature ventricular complex.

Supplementary Figure S1. Cumulative incidence of ischemic stroke according to PVC Location in OAC-naïve NVAF patients.

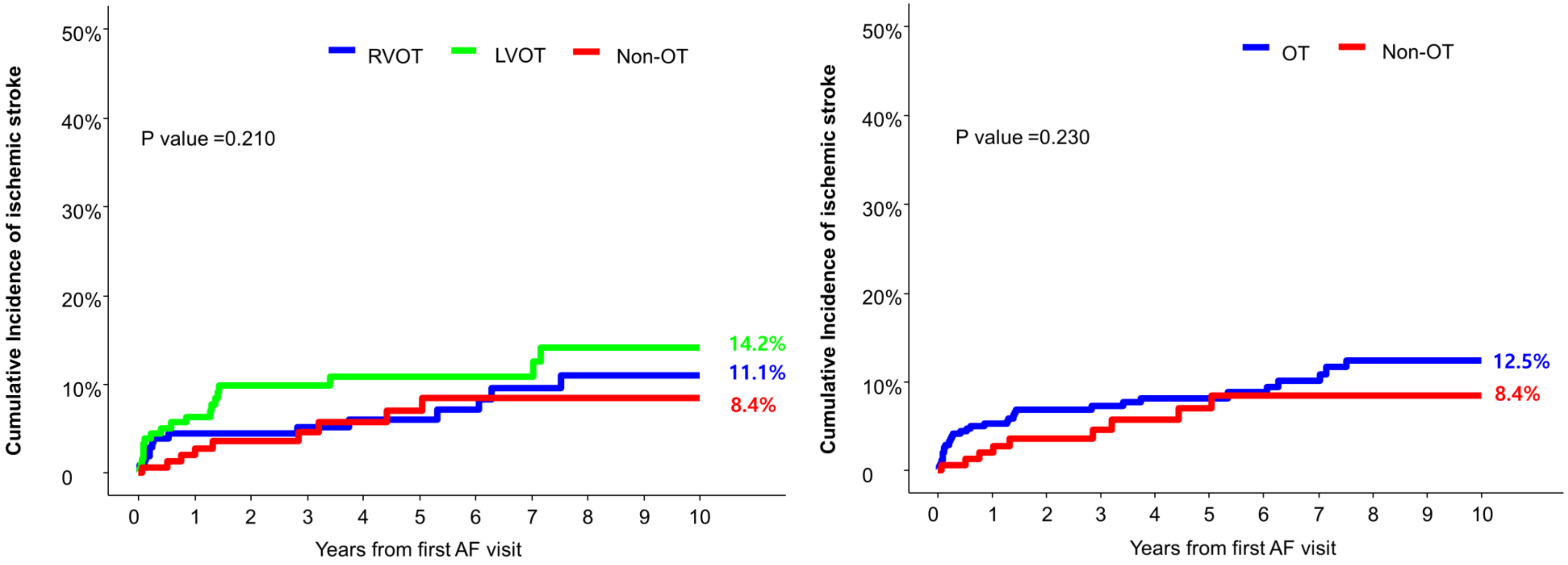

Number at risk

RVOT	212	172	144	125	104	89	80	65	44	38	31
LVOT	191	141	118	101	84	74	64	54	41	34	30
Non-OT	161	132	112	90	76	66	58	49	38	31	27

Number at risk

OT	403	313	262	226	188	163	144	119	85	72	61
Non-OT	161	132	112	90	76	66	58	49	38	31	27
